# Molecular regulation of skeletal muscle mass and the contribution of nitric oxide: A review

**DOI:** 10.1096/fba.2018-00080

**Published:** 2019-04-11

**Authors:** Jun Kobayashi, Hiroyuki Uchida, Ayaka Kofuji, Junta Ito, Maki Shimizu, Hyounju Kim, Yusuke Sekiguchi, Seiji Kushibe

**Affiliations:** ^1^ Department of Clinical Dietetics and Human Nutrition, Faculty of Pharmacy and Pharmaceutical Science Josai University Saitama Japan; ^2^ Department of Management, Faculty of Management Josai University Saitama Japan

**Keywords:** fork head box O (FoxO), mammalian target of rapamycin (mTOR), nitric oxide synthase (NOS), reactive oxygen species (ROS), ubiquitin proteasome system (UPS)

## Abstract

A variety of internal and external factors such as exercise, nutrition, inflammation, and cancer‐associated cachexia affect the regulation of skeletal muscle mass. Because skeletal muscle functions as a crucial regulator of whole body metabolism, rather than just as a motor for locomotion, the enhancement and maintenance of muscle mass and function are required to maintain health and reduce the morbidity and mortality associated with diseases involving muscle wasting. Recent studies in this field have made tremendous progress; therefore, identification of the mechanisms that regulate skeletal muscle mass is necessary for the physical and nutritional management of both athletes and patients with muscle wasting disease. In this review, we present an overall picture of the interactions regulating skeletal muscle mass, particularly focusing on the insulin‐like growth factor‐I (IGF‐I)/insulin‐Akt‐mammalian target of rapamycin (mTOR) pathway, skeletal muscle inactivity, and endurance and resistance exercise. We also discuss the contribution of nitric oxide (NO) to the regulation of skeletal muscle mass based on the current knowledge of the novel role of NO in these processes.

**Abbreviations**
AMP/ATP: adenosine monophosphate/adenosine triphosphate
AMPK: AMP‐activated protein kinase
ATGs: autophagy‐related genes
cGMP: cyclic guanosine‐monophosphate
CPT‐1: carnitine palmitoyltransferase 1
CREB1: cAMP response element‐binding protein 1
DGC: dystrophin glycoprotein complex
eNOS: endothelial nitric oxide synthase
FoxO: fork head box O
GRP94: glucose‐regulated protein 94
IGF‐I: insulin‐like growth factor‐I
MAFbx: muscle atrophy F box
mtDNA: mitochondrial DNA
mTOR: mammalian target of rapamycin
MuRF‐1: muscle ring finger 1
n/eNOS: neuronal and endothelial nitric oxide synthase
nNOSμ: neuronal nitric oxide synthase μ
nNOSμ: neuronal nitric oxide synthase μ
NO: nitric oxide
NO: nitric oxide
NRF1/2: nuclear respiratory factors 1/2
ONOO^‐^: peroxynitrite anion
PGC‐1α: peroxisome proliferator‐activated receptor gamma coactivator‐1α
PGC‐1α: peroxisome proliferator‐activated receptor‐γ coactivator 1α
PI3K: phosphoinositide 3‐kinase
PKA: protein kinase A
ROS: reactive oxygen species
RyR1: type 1 ryanodine receptor
RyR1: type 1 ryanodine receptor
SR: sarcoplasmic reticulum
SR: sarcoplasmic reticulum
TF: transcription factor
TFAM: mitochondrial transcription factor A
TFB1M: mitochondrial transcription factor B1
TFB2M: mitochondrial transcription factor B2
TFEB: transfactor EB
TRPV1: transient receptor potential vanilloid subfamily, member 1
UPS: ubiquitin proteasome system

## 1 BACKGROUND

Increasing muscle strength and maintaining muscle mass and function are necessary not only for athletes but also for aging people; rather than just as a motor for locomotion, skeletal muscle functions as a crucial regulator of whole body metabolism.^1^, ^2^ Systemic wasting conditions cause the rapid loss of muscle mass, weakness, and increased disability, consequently reducing the quality of life, and are directly linked to mortality.^3^ Recent research in this field has made tremendous progress^4^; identification of the mechanisms that regulate skeletal muscle mass is required for the physical and nutritional management of athletes and patients with muscle wasting.

Reactive oxygen species (ROS)‐ and reactive nitrogen species (RNS)‐mediated signaling processes are very important in the maintenance of skeletal muscle mass. ROS/RNS are endogenously generated from superoxide and nitric oxide (NO), respectively, at low levels under resting conditions, which become elevated during skeletal muscle contraction.^5^ While they affect the physiological functions of myofilaments, the sarcoplasmic reticulum (SR), and other cellular proteins and structures via redox‐mediated signaling pathways, a non‐physiological increase in ROS/RNS causes an imbalance between skeletal muscle protein synthesis and degradation, leading to muscle atrophy.^6^ A variety of factors beneficially or harmfully affect skeletal muscle mass though ROS/RNS‐mediated mechanisms. Therefore, increasing attention has been paid to NO‐mediated regulation of skeletal muscle mass in health and disease.^7^, ^8^

In this short review, we give an overall description of the interactions between the factors regulating skeletal muscle mass and muscle atrophy/hypertrophy, particularly focusing on the insulin‐like growth factor‐I (IGF‐I)/insulin‐Akt‐mammalian target of rapamycin (mTOR) pathway, skeletal muscle inactivity, and exercise. We also discuss the NO contribution to the regulation of skeletal muscle mass by presenting the current knowledge on the novel roles of NO in this research field.

## 2 FACTORS REGULATING SKELETAL MUSCLE MASS: THE OVERALL PICTURE

**Figure 1:**
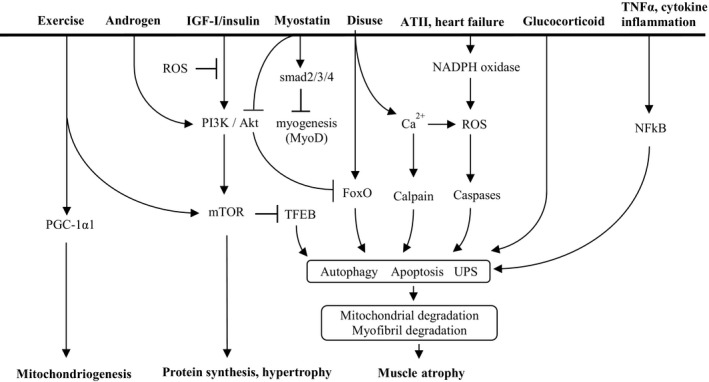
Overall scheme of the factors regulating skeletal muscle mass. The factors regulating skeletal muscle mass. There are three outcomes resulting from skeletal muscle regulation; mitochondriogenesis, hypertrophy, and atrophy. ATII, angiotensin II; FoxO, Fork head box O; IGF‐Ⅰ, insulin growth factor‐I; mTOR, mammalian target of rapamycin; NADPH oxidase, nicotinamide adenine dinucleotide phosphate oxidase; NFkB, nuclear factor kappa B; PGC‐1α1, peroxisome proliferator‐activated receptor gamma coactivator‐1α1; PI3K, phosphoinositide 3‐kinase; ROS, reactive oxygen species; TFEB, transcription factor EB; TNFα, tumor necrosis factor α; UPS, ubiquitin proteasome system

A variety of internal and external stimuli ranging from exercise training to prolonged bed rest and cancer‐associated cachexia, affect the regulation of skeletal muscle mass (Figure 1). Although the signaling pathways regulating skeletal muscle mass are complicatedly intertwined, IGF‐I/insulin signaling is a main trigger controlling skeletal muscle protein balance not only by interacting with protein kinases such as Akt and its downstream effectors, mTOR and transcription factor EB (TFEB), but also by being affected by exercise, myostatin, and anabolic hormones.

As aging progresses, protein synthesis in skeletal muscle declines (called sarcopenia), partly due to a reduction in the levels of anabolic hormones such as androgen (testosterone), growth hormone, and IGF‐I.^4^ Particularly in males, the reduced production and bioavailability of androgens during aging directly contributes to muscle atrophy because androgens play a major role in the maintenance and restoration of muscle mass by stimulating the upstream pathway of mTOR (Figure 1).^9^ Angiotensin II (ATII) is a main effector molecule of the renin‐angiotensin system, which physiologically maintains the sodium and water balance. In congestive heart failure, ATII increases ROS production in skeletal muscle via the angiotensin II type 1 receptor (AT1R) and NADPH oxidase‐dependent mechanism, which subsequently induces proteolytic enzymes leading to muscle protein degradation, and also inhibits the IGF‐I/insulin‐Akt‐mTOR pathway by phosphorylation of serine residues on the insulin receptor substrate (IRS) (Figure 1).^10^ Glucocorticoids inhibit IGF‐I‐mediated muscle growth; they stimulate myostatin expression resulting in the downregulation of protein synthesis as well as the proliferation and differentiation of muscle satellite cells (Figure 1).^11^ Inflammatory cytokines, particularly tumor necrosis factor α (TNFα), activate the transcription factor NF‐κB expressed in skeletal muscle through the activation of IκB kinase (IKKβ), which phosphorylates IκB, leading to the nuclear translocation of NF‐κB and activation of NF‐κB‐mediated gene transcription, including genes that regulate the ubiquitin proteasome system (UPS) (Figure 1).^12^, ^13^

**Figure 2:**
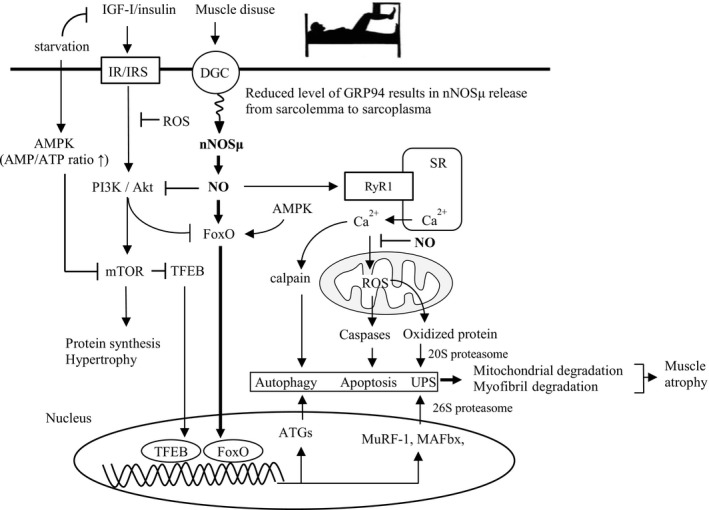
Prolonged skeletal muscle inactivity (disuse). Prolonged muscle disuse leads to nNOS‐mediated muscle atrophy via a FoxO‐dependent pathway. The stress protein, Grp94, while serving as a chaperone for folding IGFs and antioxidant cytoprotection, functions to stabilize subsarcolemmal nNOSµ. AMP/ATP, adenosine monophosphate/adenosine triphosphate; AMPK, AMP‐activated protein kinase; ATGs, autophagy‐related genes; DGC, dystrophin glycoprotein complex; FoxO, Fork head box O; GRP94, glucose‐regulated protein 94; IGR‐I, insulin‐like growth factor‐I; IR/IRS, insulin receptor/ insulin receptor substrate; MAFbx, muscle atrophy F box; mTOR, mammalian target of rapamycin; MuRF‐1, muscle ring finger 1; nNOSμ, neuronal nitric oxide synthase μ; NO, nitric oxide; ROS, reactive oxygen species; RyR1, type 1 ryanodine receptor; SR, sarcoplasmic reticulum; TFEB, transfactor EB; UPS, ubiquitin proteasome system

The above mentioned diverse catabolic stimuli, followed by the activation of key transcription factors, fork head box O (FoxO) and NFκB, and proteolytic enzymes such as calpains and caspases, result in skeletal muscle atrophy. However, the responses following these catabolic stimuli all share a set of common downstream transcriptional pathways leading to increased protein degradation and reduced protein synthesis (Figure 1). Microarray studies identified atrophy‐related genes (atrogenes) that are induced or repressed in various wasting conditions. Among these genes are those encoding several E3 ubiquitin ligases such as muscle ring finger 1 (MURF1) and muscle atrophy F box (MAFbx, also called atrogin1), which mark the target proteins by ubiquitination, and form the UPS to degrade myofibrillar proteins by the 26S proteasome.^14^ However, oxidized proteins induced by ROS (eg in heart failure) are degraded by the 20S proteasome without prior ubiquitination (Figure 2).^15^ The detailed mechanisms of how the UPS removes short‐living proteins can be found elsewhere.^16^ In contrast, the autophagy‐lysosomal system (ALS) is a proteolytic system that removes long‐living proteins such as defunct organelles and mitochondria.^17^, ^18^ The activated caspases and calpains also degrade muscle proteins directly or through the upstream UPS and ALS (Figure 2). In these processes, UPS causes reduced muscle strength through the degradation of myofibrillar proteins in the contractile machinery, and ALS causes reduced endurance capacity through degradation of the mitochondria and other organelles.^4^

Recent accumulating evidence has also shown a coordinated interaction of muscle atrophy with endoplasmic reticulum (ER) stress and the subsequent unfolded protein response (UPR).^19^ The ER orchestrates the synthesis, folding, and structural maturation of cellular proteins. However, aging, exercise, starvation, cancer cachexia, denervation, high fat diet, and many other perturbations disrupt cell homeostasis leading to ER stress. The ER deals with such stresses through initiating UPR, which properly folds the unfolded and misfolded proteins to control protein quality.^20^ In these processes, a physiological range of ER stress protects the muscle from undergoing further damage by inducing ER chaperone proteins. On the other hand, high levels of ER stress, such as cancer and heart failure‐associated cachexia, initiate the transcriptional atrophy programs leading to autophagy and apoptosis, which result in further loss of skeletal muscle mass. Because ER stress and UPR are beyond the scope of this review, readers are referred to the papers of Cybulsky,^21^ Afroze,^20^ and Bohnert^22^ for more details.

## 3 IGF‐I/INSULIN‐Akt‐MTOR PATHWAY

In the regulation of muscle growth and atrophy, the IGF‐I/insulin‐Akt‐mTOR pathway is thought to be the main regulatory mechanism. This pathway is largely influenced by physical activity, nutrition, and various diseased conditions (Figures 1, 2).^4^ The anabolic effects of IGF‐I/insulin on skeletal muscle are mediated through specific binding with IRS to promote the activation of the phosphoinositide 3‐kinase (PI3K)‐Akt‐mTOR signaling pathway. Under normal conditions, IGF‐I/insulin signaling appears to be dominant against other catalytic signaling proteins such as myostatin (called myokine and inhibitor of myogenesis), and transcription factors such as FoxO, because the nuclear translocation of FoxO and transcription factor EB (TFEB) are inhibited by Akt‐ and mTOR‐mediated phosphorylation, respectively (Figure 1). However, in case of starvation and critical illness (eg systemic inflammation and cancer cachexia), muscle metabolism represents an adaptive catabolic response characterized by enhanced muscle proteolysis and amino acid release to sustain liver gluconeogenesis and increased fatty acid oxidation to provide a major energy source by increasing the activity of lipoprotein lipases and the subcellular distribution of CD36 for fatty acid uptake by skeletal muscle cells.^23^ This is caused by decreased IGF‐I/insulin‐Akt‐mTOR signaling and the subsequent translocation of dephosphorylated FoxO and TFEB to the nucleus, followed by the enhanced transcription of atrogenes and muscle protein degradation through the UPS.

Another important factor affecting the IGF‐1/insulin‐Akt‐mTOR pathway in the regulation of skeletal muscle mass is myostatin, an extracellular cytokine (a member of transforming growth factor‐β (TGF‐β) superfamily) mostly expressed by skeletal muscle, and a potent negative regulator of muscle growth and the activation of satellite cells (stem cells resident in skeletal muscle).^24^ During embryogenesis, myostatin expression is restricted to the developing skeletal muscles. It is still expressed and secreted by skeletal muscles in adulthood, playing the roles in inhibiting cell cycle progression and reducing levels of myogenic regulatory factors, thereby controlling myoblastic proliferation and differentiation during developmental myogenesis. The PI3K‐Akt‐mTOR signaling pathway induces hypertrophy of skeletal muscle, which increases the secretion of myostatin, which then controls skeletal muscle mass by inhibiting PI3K‐Akt‐mTOR signaling.^25^ The role of myostatin in muscle wasting is mediated by activin receptor IIB (ActRIIB), which leads to several different signaling cascades. The binding of myostatin to ActRIIB phosphorylates the transcription factor smad2/3 complex, leading it to form a complex with smad4 (a tumor suppressor), resulting in nuclear translocation and blocking of the transcription of genes responsible for myogenesis, such as MyoD (Figure 1). ActRIIB‐mediated myostatin signaling also phosphorylates MAPK and activates its downstream cascade leading to the downregulation of the myogenesis genes.^25^ Furthermore, myostatin is reported to inhibit Akt signaling by suppressing the expression of miR‐486, a positive regulator of the IGF‐I/Akt pathway, resulting in the upregulation of the atrogenes, as shown in an in vitro study 26, 27 (Figure 1). Inhibition of the myostatin‐ActRIIB pathway results in muscle fiber hypertrophy, which has received much interest as a possible therapeutic strategy for a variety of muscle diseases such as cancer‐associated cachexia.^28^

## 4 PROLONGED SKELETAL MUSCLE INACTIVITY

Prolonged periods of muscle disuse result in increased ROS generation in limb skeletal muscles. Recent evidence suggests that prolonged skeletal muscle inactivity results in increased superoxide production at multiple locations in the cells including NADPH oxidase, xanthine oxidase, and in the mitochondria.^29^, ^30^, ^31^, ^32^, ^33^ In particular, the mitochondria are thought to be a major source of ROS generation in inactive skeletal muscle.^31^, ^32^ Furthermore, prolonged inactivity of limb skeletal muscle is accompanied with increased NO levels in the inactive muscle. Unlike in inflammatory and cachectic muscles, inducible NOS (iNOS) levels and iNOS‐derived NO do not increase in these disused skeletal muscles. Rather, Suzuki et al reported that neuronal NOS (nNOS) is the source of NO during prolonged skeletal muscle inactivity, suggesting a possible cause and effect relationship between increased productions of ROS and RNS in the disused skeletal muscle.^34^

The sarcolemmal localization of nNOSµ (a splice variant of nNOS) by binding to the PDZ domain (postsynaptic density protein 95/discs large/ZO‐1 homology domain) of α‐syntrophin of the dystrophin glycoprotein complex (DGC) is a critical determinant of the physiological vasoregulatory effect of skeletal muscle‐derived NO (Figure 2).^35^ The translocation of sarcolemmal nNOSµ to the sarcoplasmic zone has been reported to occur in response to various forms of skeletal muscle disuse, for example, hindlimb unloading,^34^, ^36^ space flight,^37^ denervation,^34^ bed rest,^38^ and intensive care‐associated critical illness myopathy.^39^ Dislocated nNOSµ to the sarcoplasma reduces physiological blood flow to the skeletal muscle and inappropriately increases cytoplasmic NO availability, resulting in nitrosations of Akt (which leaves FoxO dephosphorylated) and FoxO, both of which accelerate FoxO nuclear translocation followed by the enhancement of UPS.^40^ In addition, increased cytoplasmic NO also causes hypernitrosation of the type 1 ryanodine receptor (RyR1), followed by increased Ca^2+^ leakage from the SR through RyR1 due to displacement of calstabin (a stabilizer for the closed state of the RyR1 channel).^41^, ^42^ Therefore, increased cytosolic Ca^2+^ levels cause ROS generation in the mitochondria and activate proteases such as calpain and caspases, leading to myofibrillar degradation and/or apoptosis (Figure 2).

On the other hand, NO inhibits calpain‐mediated skeletal muscle proteolysis via *S*‐nitrosation of the cysteine residue of calpain, as shown in an in vitro study.^43^, ^44^ In skeletal muscles, there are high concentrations of potent NO scavengers including myoglobin and glutathione, which could therefore limit diffusion‐based NO signaling and localize cGMP‐dependent and independent signaling effects in the close vicinity of target proteins. Whether NO plays a protective or detrimental role in muscle mass regulation depends on its quantitative effect and spatial distribution in the skeletal muscle cells. When considering dietary and pharmacological interventions, L‐arginine or citrulline supplementation as substrates for nNOS and eNOS would favorably enhance NOS activity where it is normally located.^45^

Although the detailed mechanism by which skeletal muscle disuse induces dislocation of sarcolemmal nNOSµ to the cytoplasm has not yet been elucidated, recent reports have shown a novel view point on this mechanism, suggesting that glucose‐regulated protein 94 (Grp94), a heat shock protein (HSP)/Ca^2+^ binding chaperone, which is closely associated with IGF folding and secretion in skeletal myogenic cells, interacts with nNOSμ and stabilizes it by preventing its untethering from the sarcolemma, as well as protecting nNOSμ from degradation by UPS.^46^ Chronic exercise training in mice (treadmill training 1 h/d, 5 d/wk for 4 weeks), has been shown to significantly increase the muscular levels of Grp94, helping to keep nNOSμ in the sarcolemma.^47^ However, the muscular levels of Grp94 significantly decrease in the unloaded myofibrils, which is associated with enhanced sarcoplasmic NO production, and subsequent initiation of the atrophying process of skeletal muscle as mentioned above.^40^, ^48^

The skeletal muscle adapts to the stress of contractile activity via changes in gene expression to yield an increased content of HSPs, including Grp94. Therefore, reduced stress response proteins due to skeletal muscle inactivity might be fundamentally related to the initiation of the tissue remodeling processes including muscle atrophy.^49^

## 5 EXERCISE

### 5.1 Exercise leading to mitochondrial biogenesis

Exercise is a potent stimulus to preserve skeletal muscle mass, aerobic fitness, and strength.^50^ In skeletal muscle contractions, action potentials generated at the neuromuscular junction depolarize rapidly along the surface membrane of the muscular fiber, propagating down the transverse tubules (T‐tubules) and activate voltage‐gated Ca^2+^ channels (L‐type Ca^2+^ channels). Voltage‐induced conformational changes of L‐type Ca^2+^ channels activate the closely apposed Ca^2+^ release channel, RyR1, on the terminal cisternae of the SR, resulting in a rapid increase in the amount of cytosolic Ca^2+^, which leads to muscle contraction through the sliding of actin and myosin filaments.^51^

**Figure 3:**
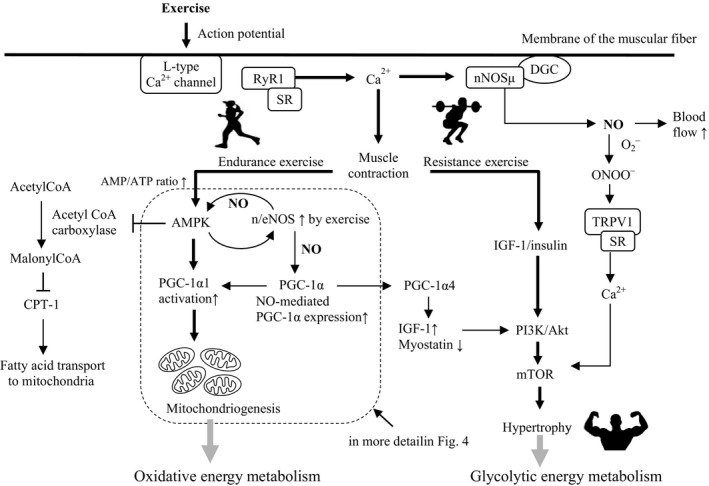
Exercise leads to mitochondrial biogenesis and muscle hypertrophy. Schematic diagram showing the two types of exercise and their downstream signaling pathways leading to mitochondriogenesis and muscle hypertrophy. AMP/ATP, adenosine monophosphate/adenosine triphosphate; AMPK, AMP‐activated protein kinase; CPT‐1, carnitine palmitoyltransferase 1; CREB1, cAMP response element‐binding protein 1; DGC, dystrophin glycoprotein complex; eNOS, endothelial nitric oxide synthase; IGF‐I, insulin‐like growth factor‐I; mTOR, mammalian target of rapamycin; n/eNOS, neuronal and endothelial nitric oxide synthase; nNOSμ, neuronal nitric oxide synthase μ; NO, nitric oxide; ONOO^‐^, peroxynitrite anion; PGC‐1α, peroxisome proliferator‐activated receptor gamma coactivator‐1α; PI3K, phosphoinositide 3‐kinase; PKA, protein kinase A; RyR1, type 1 ryanodine receptor; SR, sarcoplasmic reticulum; TRPV1, transient receptor potential vanilloid subfamily, member 1

There are two types of exercise that affect the skeletal muscle phenotype, endurance and resistance exercise; skeletal muscle adapts to the type of exercise by transforming the phenotype to include mainly slow or fast twitch fibers.^52^ In addition, these two types of exercise are contrary to each other in terms of subsequent muscle phenotype expression.^53^ Hickson demonstrated that endurance exercise after resistance exercise (endurance + resistance) had detrimental effects on muscle strength gain compared to resistance exercise alone,^54^ suggesting a training interference between the two, which might be caused by some metabolic conversion during the exercises.^55^ Recent studies reported that peroxisome proliferator‐activated receptor‐γ coactivator 1α (PGC‐1α) plays a very important role in the phenotypic differentiation of skeletal muscle. Endurance exercise induces fast to slow muscle phenotype transformation, mitochondrial biogenesis, and angiogenesis through the AMP‐activated protein kinase (AMPK)‐PGC‐1α1 pathway.^52^ On the other hand, resistance exercise stimulates muscle protein synthesis leading to hypertrophy via the Akt‐mTOR‐mediated pathway, which is accompanied by the increased expression of PGC‐1α4, an alternatively spliced transcript of the PGC‐1α parent gene (Figure 3).^52^, ^56^ A series of studies on this issue suggest that there may be a molecular switch mediated by exercise intensity, the so‐called AMPK‐Akt master switch, which partially mediates specific adaptations to endurance or resistance exercise.^57^

**Figure 4:**
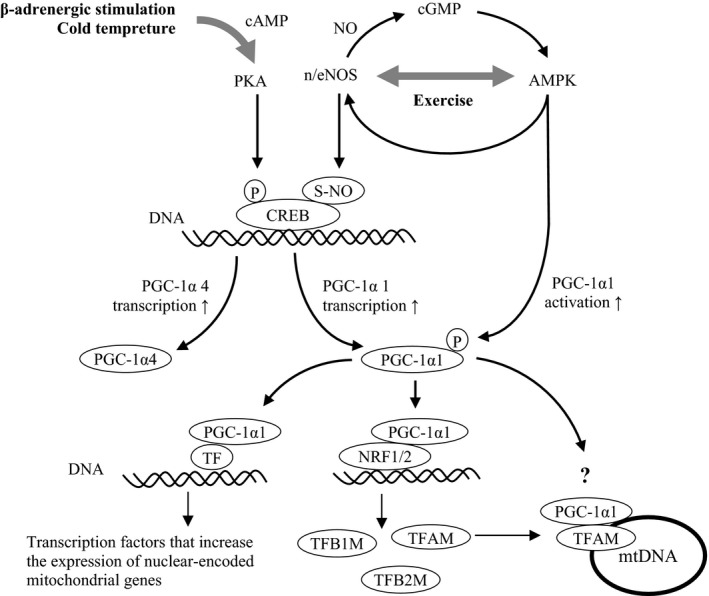
PGC‐1α, a master regulator of mitochondrial biogenesis. NO‐mediated transcription and activation of PGC‐1α. PGC‐1α is a master regulator of mitochondrial biogenesis. AMPK, AMP‐activated protein kinase; cGMP, cyclic guanosine‐monophosphate; CREB, cAMP response element‐binding protein 1; mtDNA, mitochondrial DNA; n/eNOS, neuronal/endothelial nitric oxide synthase; NRF1/2, nuclear respiratory factors 1/2; PGC‐1α, peroxisome proliferator‐activated receptor‐γ coactivator 1α; PKA, protein kinase A; TF, transcription factor; TFAM, mitochondrial transcription factor A; TFB1M, mitochondrial transcription factor B1; TFB2M, mitochondrial transcription factor B2

During endurance exercise, AMPK is allosterically activated via an increased AMP/ATP ratio and is phosphorylated via a Ca^2+^ dependent signaling pathway, followed by phosphorylation of PGC‐1α1, and the subsequent activation of a number of transcriptional factors associated with mitochondrial biogenesis and angiogenesis including nuclear respiratory factor 1 and 2 (NRF1/2), peroxisome proliferator‐activated receptor (PPARδ), estrogen‐related receptor (ERRα), and myocyte enhancer factor 2 (MEF2). Activated AMPK also phosphorylates acetyl‐CoA carboxylase (ACC), then reduces the concentration of malonyl‐CoA by inhibiting ACC, which in turn reduces the allosteric inhibition of carnitine palmitoyltransferase 1 (CPT‐1).^58^ This allows increased rates of fat oxidation following fatty acid transport into the mitochondria to provide the major energy source in endurance exercise (Figure 3, and in more detail in Figure 4).

NO is closely associated with the AMPK‐PGC‐1α1‐mediated signaling process by cooperatively modulating muscle contraction and oxidative metabolism,^59^ contributing to the mitochondrial biogenesis of skeletal muscle (Figure 4).^8^ In endurance exercise, NO produced by nNOS (and eNOS) not only enhances blood flow and oxygen delivery to efficiently meet the metabolic demands of active muscle,^35^ but also enhances cGMP‐mediated AMPK activation and PGC‐1α1 phosphorylation.^60^ NO‐induced AMPK activation also phosphorylates nNOS and eNOS, forming a positive feedback activation between AMPK and NOS (Figure 4). A number of reports have shown the NO implication in AMPK activation and subsequent mitochondrial biogenesis.^59^ Exercise and cold temperature‐mediated stimuli to the sympathetic nervous system via β‐adrenergic receptors enhance the transcription of PGC‐1α (mainly another alternatively spliced variant PGC‐1α4) via the protein kinase A (PKA)/cAMP response element‐binding protein 1 (CREB1) pathway (Figure 4).^8^, ^61^ In addition, *S*‐nitrosation of CREB by NO more efficiently engages with the promotor of the genes encoding PGC‐1α1, exhibiting a stronger transcriptional induction of PGC‐1α1 (Figure 4).

Thus, increased transcription and activation of PGC‐1α1 interact with NRF1/2, inducing the expression of nucleus‐encoded mitochondrial genes including mitochondrial transcription factor A (TFAM) and proteins involved in oxidative phosphorylation. PGC‐1α1 also interacts with TFAM on mitochondrial DNA (mtDNA) and coactivates the transcription of mitochondria‐encoded mitochondrial genes (Figure 4).^7^, ^8^, ^62^

### 5.2 Exercise leading to muscle hypertrophy

According to the study by Chen et al,^63^ AMPK activation following exercise is intensity‐dependent; intensities of 60% VO_2_max have been consistently reported to induce AMPK activation. However, during exercise at intensities greater than approximately 60% VO_2_max, blood glucose and muscle glycogen become the primary fuels oxidized to produce the ATP required to sustain exercise, resulting in an increased reliance on carbohydrates as the predominant fuel source. Therefore, high intensity exercise including resistance exercise stimulates glycogenolysis, and subsequently the Akt‐mTOR pathway, leading to skeletal muscle hypertrophy.^2^

NO is reported to contribute to exercise‐induced muscle hypertrophy. Myotubule depolarization induces physiological superoxide generation through the activation of NADPH oxidase,^64^ which is localized to the sarcolemma and T‐tubules. The increasing frequency of contractions enhances the activity of skeletal muscle NADPH oxidase with increasing superoxide generation. A potential physiological role for this process was proposed by Hidalgo et al who suggested that the superoxide generated by NADPH oxidase can stimulate Ca^2+^ release from the SR through oxidative modification of the RyR1.^65^ Muscular contraction and exercise generate an increased production of ROS and NO.^66^ Unlike the large amount of pathological ROS production by the mitochondria, exercise‐induced ROS is primarily of non‐mitochondrial origin, particularly from NADPH oxidase.^67^, ^68^ The increase in intracellular Ca^2+^ that occurs upon muscle excitation increases nNOS (Ca^2+^/calmodulin‐activated enzyme) activity several fold during contraction. NO and peroxynitrite then activate the transient receptor potential vanilloid subfamily, member 1 (Trpv1, also known as capsaicin receptor), resulting in an increase in the intracellular Ca^2+^ concentration that subsequently triggers activation of the protein kinase mTOR (Figure 3).^69^

One final pathway that modulates skeletal muscle mass following exercise involves resistance exercise‐induced muscle hypertrophy via the PGC‐1α4‐mediated pathway. As mentioned above, this pathway is thought to be activated by resistance exercise‐induced β‐adrenergic stimulation, followed by alternative splicing of the PGC‐1α parent gene to express PGC‐1α4 rather than PGC‐1α1 (Figure 4).^56^, ^70^ A study by Ruas and his colleagues showed that myotubule hypertrophy was induced by treatment with the β‐adrenergic agonist with robust hypertrophy accompanied by a 5‐fold increase in endogenous PGC‐1α4 levels and 1.9‐fold increase in the protein/DNA ratio. However, no changes were observed in PGC‐1α1 levels with β‐adrenergic agonist treatment. These results show that PGC‐1α4 is required for myotubule hypertrophy in their cellular model.^56^ Popov et al also suggested that PGC‐1α4 expression may be promoted through β2 adrenergic receptor signaling to the alternative promotor gene (Figure 4).^71^ PGC‐1α4 regulates genes in the IGF‐I and myostatin pathways (increased and reduced expression of IGF‐I and myostatin, respectively), which are well‐known to regulate skeletal muscle hypertrophy (Figure 3). Although these results are notable, the proposal that skeletal muscle hypertrophy following resistance exercise is mediated through PGC‐1α4 remains a matter of debate,^72^ and requires further investigation in future studies.^73^

### 5.3 Therapeutic application of NO in skeletal muscle wasting and exercise performance

As mentioned above, NO is involved in the regulation of skeletal muscle mass; therefore, it is natural to consider the therapeutic application of NO in the prevention of skeletal muscle wasting. NO is associated with skeletal muscle atrophy in muscle disuse, and inflammation and cachexia due to the intracellular spatial arrangement and quantitative effect of NO synthesis by dislocated nNOS and cytokine‐activated iNOS, respectively.^34^ Thus far, there has been no report on a therapeutic strategy of pharmacological and nutritional NO donors to prevent muscle wasting in these conditions. However, reactive nitrogen intermediates (RNIs) including NO play an important role in regulating energy metabolism in skeletal muscle mitochondria.^74^ RNIs and NO elicit cytoprotective effects at several levels of the mitochondrial respiratory process (eg cytochrome c oxidase and complex I), particularly through inhibition of the electron transport chain and subsequent ROS production after ischemia‐reperfusion injury.^74^

On the other hand, exercise induces NO production and stimulates NO‐mediated signaling processes leading to mitochondrial biogenesis and skeletal muscle cell hypertrophy. Larsen recently showed that dietary nitrate, which is metabolized to nitrite, NO, and other RNIs via the enterosalivary nitrate‐nitrite‐NO pathway, improves mitochondrial respiratory efficiency in human skeletal muscle, which is coupled to reduced proton leak across the uncoupling protein‐3 (UCP‐3) expressed in the inner mitochondrial membrane and a reduction in the expression of adenine nucleotide translocase (ANT).^74^ This oxygen‐sparing effect of dietary nitrate supplementation is associated with reduced oxygen cost during exercise, leading to exercise tolerance. These findings have been confirmed in many human studies using beetroot juice as the source of nitrate,^74^, ^75^ which may provide a promising nutritional strategy for promoting skeletal muscle performance.

## 6 CONCLUSIONS

Advancing age, low physical activity, malnutrition, inflammation, and cancer‐associated cachexia all account for skeletal muscle wasting, which substantially reduces the quality of life. In the regulation of skeletal muscle mass, NO is a key signaling molecule and plays an important role in skeletal muscle physiology because it is tightly linked to many relevant pathways (AMPK, PGC‐1α, and PI3K/Akt‐mTOR signaling pathways) in the maintenance of both skeletal muscle integrity and proper signaling mechanisms during adaptation to mechanical and metabolic stimulation. On the other hand, the inappropriate distribution and increased generation of NO due to muscle inactivity and inflammation result in the non‐physiological production of ROS and RNS, leading to the catabolic responses and muscle atrophy via UPS and autophagy. When considering NO biology and its therapeutic application in the clinical setting, it is important that this freely diffusible, highly reactive radical is short‐lived, particularly in skeletal muscle where NO scavengers such as myoglobin and glutathione are in close vicinity to newly generated NO. Therefore, NO bioavailability, whether beneficial or harmful,^76^ is greatly affected by its concentration and distribution. NO bioavailability can depend on how it is administered, such as diet (vegetables rich in nitrate),^77^ supplements (nitrite/nitrate, and L‐arginine/citrulline as the substrates for NOSs),^78^, ^79^ and drugs (NO donors).^43^, ^59^, ^80^, ^81^ As it becomes clearer that NO participates in the regulation of skeletal muscle mass, future studies should focus on NO‐mediated therapeutic applications in health and disease.

## CONFLICT OF INTEREST

No conflicts of interest, financial or otherwise, are declared by the authors.

## AUTHOR CONTRIBUTIONS

J. Kobayashi designed the study, and wrote the initial draft of the manuscript. H. Uchida, A. Kofuji, J. Ito, M. Shimizu, K. Hyounju, Y. Sekiguchi, and S. Kushibe assisted in the preparation of the manuscript and also have contributed to data collection and interpretation, and critically reviewed the manuscript. All authors approved the final version of the manuscript, and agree to be accountable for all aspects of the work in ensuring that questions related to the accuracy or integrity of any part of the work are appropriately investigated and resolved.
